# Incidence and Prevalence of Chronic Obstructive Pulmonary Disease among Aboriginal Peoples in Alberta, Canada

**DOI:** 10.1371/journal.pone.0123204

**Published:** 2015-04-13

**Authors:** Maria B. Ospina, Don Voaklander, Ambikaipakan Senthilselvan, Michael K. Stickland, Malcolm King, Andrew W. Harris, Brian H. Rowe

**Affiliations:** 1 School of Public Health, University of Alberta, Edmonton, Alberta, Canada; 2 Division of Pulmonary Medicine, Faculty of Medicine & Dentistry, University of Alberta, Edmonton, Alberta, Canada; 3 Faculty of Health Sciences; Simon Fraser University, Vancouver, British Columbia, Canada; 4 Alberta Health, Edmonton, Alberta, Canada; 5 Department of Emergency Medicine, Faculty of Medicine & Dentistry, University of Alberta, Edmonton, Alberta, Canada; National Institute of Health, ITALY

## Abstract

**Background:**

Chronic obstructive pulmonary disease (COPD) is a major respiratory disorder, largely caused by smoking that has been linked with large health inequalities worldwide. There are important gaps in our knowledge about how COPD affects Aboriginal peoples. This retrospective cohort study assessed the epidemiology of COPD in a cohort of Aboriginal peoples relative to a non-Aboriginal cohort.

**Methods:**

We used linkage of administrative health databases in Alberta (Canada) from April 1, 2002 to March 31, 2010 to compare the annual prevalence, and the incidence rates of COPD between Aboriginal and non-Aboriginal cohorts aged 35 years and older. Poisson regression models adjusted the analysis for important sociodemographic factors.

**Results:**

Compared to a non-Aboriginal cohort, prevalence estimates of COPD from 2002 to 2010 were 2.3 to 2.4 times greater among Registered First Nations peoples, followed by the Inuit (1.86 to 2.10 times higher) and the Métis (1.59 to 1.67 times higher). All Aboriginal peoples had significantly higher COPD incidence rates than the non-Aboriginal group (incidence rate ratio [IRR]: 2.1; 95% confidence interval [CI]: 1.97, 2.27). COPD incidence rates were higher in First Nation peoples (IRR: 2.37; 95% CI: 2.19, 2.56) followed by Inuit (IRR: 1.92; 95% CI: 1.64, 2.25) and Métis (IRR: 1.49; 95% CI: 1.32, 1.69) groups.

**Conclusions:**

We found a high burden of COPD among Aboriginal peoples living in Alberta; a province with the third largest Aboriginal population in Canada. Altogether, the three Aboriginal peoples groups have higher prevalence and incidence of COPD compared to a non-Aboriginal cohort. The condition affects the three Aboriginal groups differently; Registered First Nations and Inuit have the highest burden of COPD. Reasons for these differences should be further explored within a framework of social determinants of health to help designing interventions that effectively influence modifiable COPD risk factors in each of the Aboriginal groups.

## Introduction

Chronic obstructive pulmonary disease (COPD) is a respiratory disorder largely caused by smoking and characterized by progressive, not fully reversible airway obstruction, systemic manifestations, and increasing frequency and severity of exacerbations [1]. Worldwide estimates of COPD prevalence are in the range of 5% to 10% [2], whereas COPD incidence rates have shown variations between 2 to 6 cases per 1,000 person-years, depending on the case definition and the study population [3].

Aboriginal peoples of Canada (First Nations peoples, Métis and Inuit) are particularly affected by respiratory diseases; the epidemiology of their respiratory problems closely mimics that of populations in many low-and middle-income countries. There are important gaps in our knowledge about the burden of COPD in Aboriginal peoples relative to their non-Aboriginal counterparts [4]. Analyses of community surveys [5] and administrative health data [6–8] have suggested that Aboriginal peoples in Canada have higher prevalence and incidence of COPD compared to non-Aboriginal populations. These studies, however, have used cross-sectional designs with self-reporting of physician diagnoses, spanned relatively short periods of observation, and limited their scope to specific Aboriginal groups (i.e., Registered First Nations or Métis only).

This is the first large, longitudinal, cohort study that evaluated the epidemiology of COPD in all three Aboriginal groups in Canada. Using a retrospective cohort design covering eight years of administrative health data from Alberta (Canada), we assessed the prevalence and incidence of COPD in the three Aboriginal groups of Registered First Nations peoples, Métis and Inuit relative to a non-Aboriginal population in the province while controlling for the potential impact of sociodemographic factors.

## Materials and Methods

### Study setting and data sources

Alberta is a culturally diverse province located in western Canada with a population of over 4 million residents, of which approximately 6.2% report Aboriginal ancestry [9]. A total of 220,695 Aboriginal people lived in Alberta by 2011, representing 15.8% of the total Aboriginal population in Canada [9]. Of these, approximately 52% are First Nations peoples, 45% are Métis and less than 1% are Inuit [9].

We obtained de-identified individual-level, longitudinal data by fiscal year (April 1^st^ of a given year to March 31^st^ of the subsequent year) from 2002 to 2010 from administrative health databases in Alberta that included records of all individuals eligible for coverage (~99% of the total population) under the publicly-funded Alberta Health Care Insurance Plan (AHCIP). Health premiums of Registered First Nations and Inuit are paid by the Canadian federal government, whereas Métis do not have special federal coverage of health services provided in the province.

Administrative health databases contained demographic information (AHCIP population registry), data on all acute and elective hospital discharges using the International Classification of Diseases, 10^th^ Revision; enhanced Canadian version (ICD-10-CA) for diagnosis coding [10] (Morbidity and Ambulatory Care Reporting System), claims for services provided by fee-for-service physicians and physicians paid under alternate payment plans with diagnostic fee codes based on the International Classification of Diseases, 9th Revision (ICD-9) [11] (Alberta Physician Claims Assessment System), and deaths that occur within the province (Alberta Vital Statistics). Additionally, the Métis Nation of Alberta (MNA) Identification Registry includes citizenship information for Métis members.

Individual records were linked across datasets based on an encrypted unique personal health number. Deterministic data linkage was used across administrative health databases, whereas probabilistic linkage was used to link data from the MNA registry with the other datasets.

### Ethics Statement

Ethics approval was obtained from the University of Alberta’s Health Research Ethics Board (HREB), in Edmonton, Alberta (Canada) (MS2_Pro 00010415). Individual patient consent was not required; however, patient records/information were anonymized and de-identified prior to analysis.

### Study population

The eligibility criteria for this study were individuals with constant registration in the AHCIP from fiscal years 2002 to 2010, who were at least 35 years of age at the beginning of each year. For the definition of the study cohorts, Aboriginal peoples were individuals with an alternate premium arrangement in the AHCIP registry (Registered First Nations, and Inuit) or, individuals identified in the MNA identification registry as Métis. Non-Aboriginals were individuals in the AHCIP registry without an alternate premium arrangement field, and not included in the MNA registry. First Nations without registration under the Indian Act [12] and Métis not included in the MNA registry were considered part of the non-Aboriginal population as there is no reliable method to identify them within the general population [13].

All Métis and Inuit in the AHCIP/population registry who met the eligibility criteria were included in the Aboriginal cohort. Random samples of eligible Registered First Nations and non-Aboriginals were selected from the AHCIP/population registry for a ratio of five Registered First Nations and five non-Aboriginals per Métis included. Cohort matching by age or sex was not considered because it was unlikely that it would improve study efficiency.

### Identification of COPD cases

**Fig 1:**
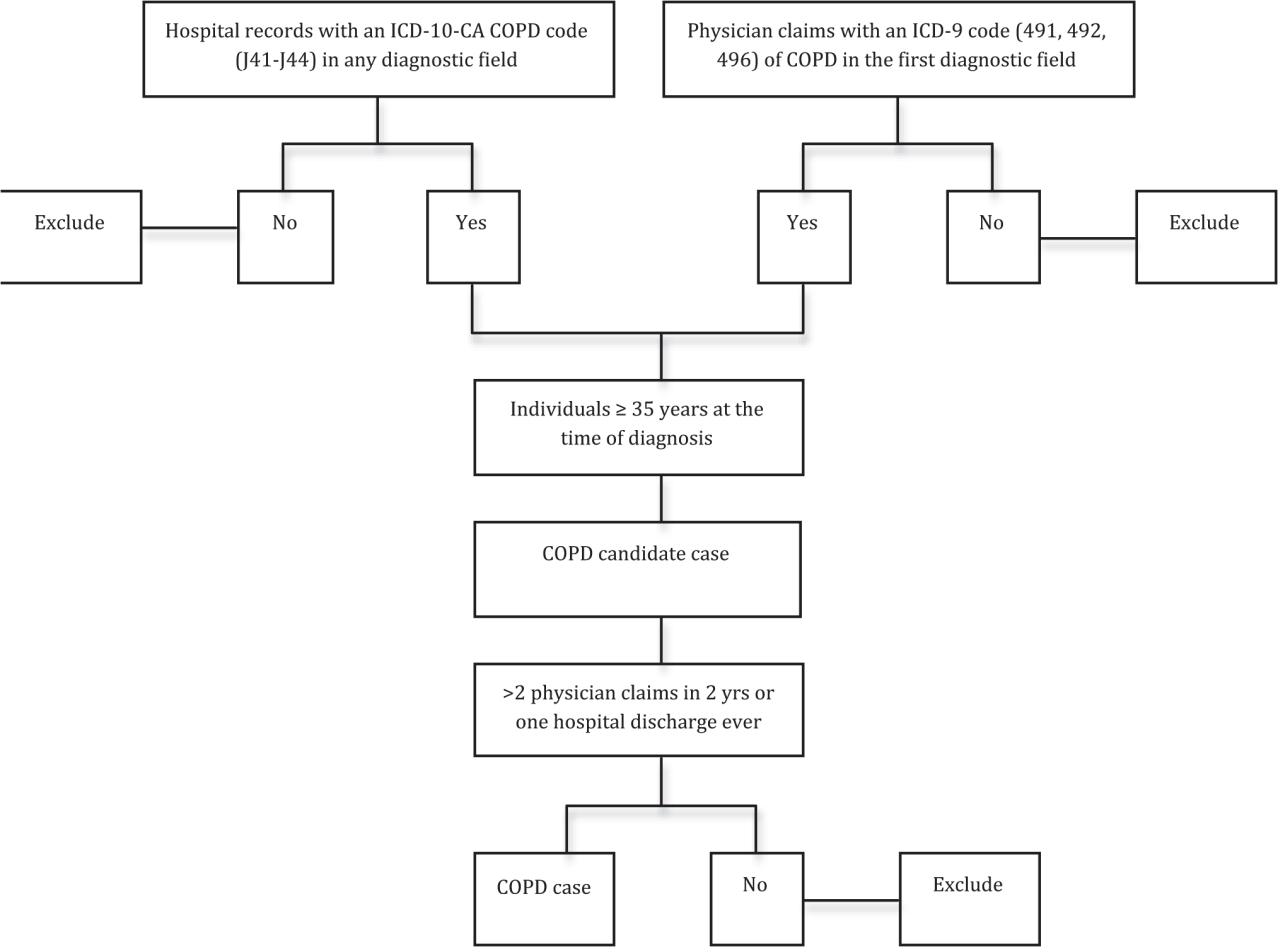
Algorithm for COPD case identification in administrative databases.

We used a validated algorithm to identify COPD cases in our study population from administrative data (Fig 1): individuals aged 35 years and older at the time of diagnosis who have at least two physician claims with an ICD-9 code (491, 492, 496) of COPD in the first diagnostic field in a two-year period, or one recording of an ICD-10-CA code (J41-J44) of COPD in any diagnostic field in the hospital discharge abstract ever, whichever came first. The two physician claims must have been on different days. When the case definition was met by two physician claims, the date of diagnosis was the date of the second physician claim [14]. This COPD algorithm has been previously validated with sensitivity of 68.4%, specificity of 93.5%, positive predictive value of 79.2%, and negative predictive value of 89.1% [15]. The index date was the date of diagnosis of COPD.

### Statistical Methods

Annual COPD prevalence for the study cohorts was calculated from fiscal years 2002 to 2010. For each fiscal year, the numerator was the number of active (alive) COPD cases from the previous fiscal year plus the new COPD cases detected by the end of the fiscal year. The denominator was the population at the beginning of the fiscal year minus ½ the total number of deaths that occurred in that year. Historical information on COPD diagnoses was collected from April 1, 1994 onwards to allow an eight-year run-in period to identify prior prevalent COPD cases present at the start of the study period [16]. Prevalence estimates were expressed as percentages.

Annual COPD incidence rates in the study cohorts were calculated from fiscal years 2002 to 2010. The numerator was the number of new COPD cases per year and the denominator was the person-time of observation (the sum of time that each person remained under risk and disease-free until COPD diagnosis, death, or end of fiscal year; whichever came first).

Incidence density rates were calculated as the total number of new COPD cases that occurred between fiscal years 2002 to 2010 divided by total person-time of observation (the sum of the time that each person remained under risk and free from disease until COPD diagnosis, death, or end of study; whichever occurred first). All incidence rates were expressed as COPD cases per 1,000 person-years.

We obtained information from the AHCIP registry on study covariates (e.g., sex, age, area of residence and socioeconomic status [SES]). Age was grouped into five 10-year intervals (35–44 years, 45–54 years, 55–64 years, 67–74 years, and 75 years and over). Area of residence was classified into urban, rural, and remote. Health care premiums in Alberta are full or partially subsidized for individuals qualifying for social assistance. As there is no direct measure of SES reported in administrative health databases in Canada, the need for, and receipt of health care subsidies (full, partial, none) is considered a proxy measure of SES within the Canadian health care system [17].

We adjusted all COPD estimates by age and sex using the direct standardization method [18] and the 1991 Canadian Census population as reference [19]. Unadjusted prevalence ratios (PR) per fiscal year were calculated. Poisson regression models were fitted for each fiscal year to adjust PRs for covariates at baseline (sex, age, area of residence and SES). Unadjusted incidence rate ratios (IRR) were calculated for every fiscal year and for the entire study period. Poisson regressions adjusted the IRRs by covariates at baseline, using person-time as the offset in the models. The non-Aboriginal cohort was the reference category in all analyses. All prevalence and incidence estimates were reported with 95% confidence intervals (CI), and two-sided p-values less than 0.05 represented statistical significance. Statistical analyses were performed using Predictive Analysis Software Statistics for Mac (PASW version 18.0, IBM SPSS, Somers NY).

### Role of funding source

The sponsors of the study had no role in the design, data collection, analysis, data interpretation, or writing of the manuscript. The corresponding author had full access to all the study data and had final responsibility for the decision to submit for publication. The opinion, results and conclusions reported in this paper are those of the authors, and independent from funding sources.

## Results

**Fig 2:**
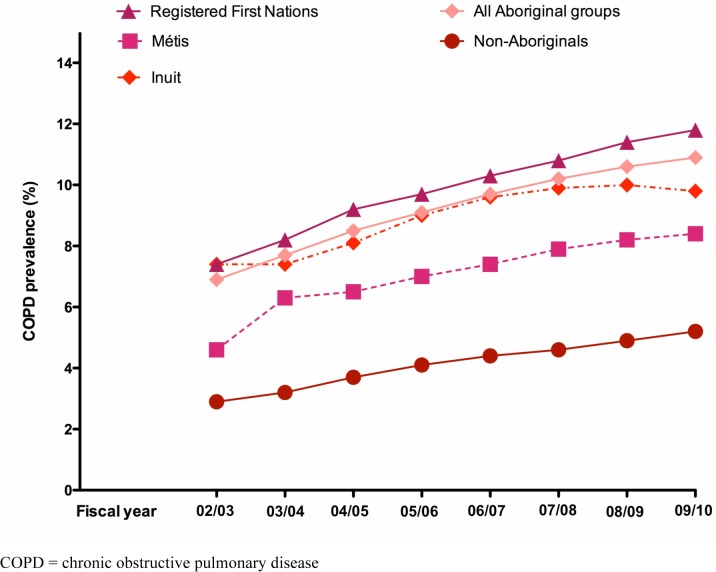
Annual age- and sex-standardized COPD prevalence rates in Aboriginal peoples and the non-Aboriginal population in Alberta, Canada from fiscal years 2002 to 2010.

A total of 79,824 individuals were followed-up over the 8-year study period (Registered First Nations [n = 32,805], Inuit [n = 1,679] Métis [n = 7,273] and non-Aboriginals [n = 38,067). (Table 1). Table 2 provides a description of the number of study participants and the number of COPD prevalent cases per year. Standardized annual COPD prevalence estimates were higher in the three Aboriginal groups than in the non-Aboriginal group, with Registered First Nations and the Inuit having the highest COPD prevalence followed by the Métis (Fig 2). Unadjusted PRs indicated that compared to the non-Aboriginal group, Aboriginal peoples had significantly higher annual COPD prevalence in every fiscal year (Table 3), particularly in Registered First Nations and Inuit groups but not in the Métis.

**Table 1:** Characteristics of the study cohorts, fiscal years 2002 to 2010.

	2002/2003	2003/2004	2004/2005	2005/2006	2006/2007	2007/2008	2008/2009	2009/2010
Total population at baseline	63,274	65,606	67,968	70,503	72,903	75,265	77,548	79,824
Aboriginal groups (%)	48.7	49.3	49.8	50.4	50.9	51.5	51.9	52.3
Registered First Nations (%)	36.9	37.5	38.1	38.8	39.4	40.1	40.6	41.1
Métis (%)	9.4	9.4	9.4	9.3	9.3	9.2	9.2	9.1
Inuit (%)	2.4	2.4	2.3	2.3	2.2	2.2	2.1	2.1
Non-Aboriginal (%)	51.3	50.7	50.2	49.6	49.1	48.5	48.1	47.7
Male (%)	49.2	49.2	49.2	49.2	49.1	49.1	49.1	49.1
Age (yr) (SD)	51.3 (12.7)	51.7 (12.8)	52.1 (13.0)	52.5 (13.2)	52.9 (13.4)	53.3 (13.5)	53.7 (13.7)	54.2 (13.9)
Age groups (%)								
35–44 yr	39.2	38.0	36.7	35.4	34.2	32.7	31.4	30.0
45–54 yr	28.3	28.5	28.9	29.3	29.5	29.8	29.9	29.9
55–64 yr	16.4	16.7	17.0	17.2	17.5	18.0	18.5	19.1
65–74 yr	9.9	10.0	10.1	10.4	10.5	10.6	10.8	11.0
≥ 75 yr	6.2	6.8	7.3	7.7	8.3	8.9	9.4	10.0
Area of residence (%)								
Urban	60.8	60.6	60.8	60.5	60.7	60.6	60.7	60.6
Rural	30.6	30.7	30.6	30.8	30.6	30.7	30.6	30.6
Remote	8.6	8.6	8.7	8.7	8.7	8.7	8.7	8.8
Subsidy level (%)								
Full	15.9	15.5	15.5	23.5	23.9	24.2	23.9	23.7
Partial	1.5	1.5	1.7	0.7	0.2	0.5	0.5	0.5
None	82.6	83.0	82.8	75.8	75.9	75.3	75.6	75.8

COPD = chronic obstructive pulmonary disease; SD = standard deviation; yr = year(s)

**Table 2:** Study population and number of prevalent cases of COPD per year in Aboriginal and non-Aboriginal cohorts.

Fiscal Year	Registered First Nations		Métis		Inuit		Non-Aboriginal	
	N	COPD prevalent cases	N	COPD prevalent cases	N	COPD prevalent cases	N	COPD prevalent cases
**2002/2003**	23,329	886	5,952	158	1,530	106	32,463	771
**2003/2004**	24,621	1,063	6,159	192	1,546	123	33,280	915
**2004/2005**	25,938	1,266	6,358	228	1,567	142	34,105	1,115
**2005/2006**	27,415	1,455	6,557	271	1,589	170	34,942	1,322
**2006/2007**	28,782	1,638	6,747	316	1,650	193	35,764	1,469
**2007/2008**	30,179	1,859	6,927	354	1,643	212	36,516	1,652
**2008/2009**	31,477	2,090	7,114	390	1,661	230	37,296	1,858
**2009/2010**	32,805	2,324	7,273	439	1,679	245	38,067	2,064

COPD = chronic obstructive pulmonary disease

**Table 3:** Annual unadjusted and adjusted prevalence ratios (PR) of COPD among Aboriginal groups in Alberta (Canada) from fiscal years 2002 to 2010.

Fiscal year	All aboriginal groups		First Nations		Métis		Inuit	
	COPD PR (95% CI)		COPD PR (95% CI)		COPD PR (95% CI)		COPD PR (95% CI)	
	Unadjusted	Adjusted	Unadjusted	Adjusted	Unadjusted	Adjusted	Unadjusted	Adjusted
2002–2003	1.57	2.13	1.59	2.31	1.11	1.60	2.91	2.10
	(1.43, 1.72)**	(1.92, 2.36)**	(1.45, 1.76)**	(2.06, 2.58)**	(0.94, 1.32)	(1.34, 1.91)**	(2.38, 3.57)**	(1.70, 2.59)**
2003–2004	1.48	2.12	1.50	2.30	1.04	1.59	2.85	2.04
	(1.36, 1.60)**	(1.92, 2.33)**	(1.38, 1.64)**	(2.08, 2.54)**	(0.89, 1.22)	(1.35, 1.87)**	(2.37, 3.42)**	(1.68, 2.48)**
2004–2005	1.44	2.12	1.46	2.32	1.03	1.61	2.70	1.98
	(1.34, 1.55)**	(1.94, 2.32)**	(1.35, 1.58)**	(2.11, 2.55)**	(0.89, 1.19)	(1.39, 1.86)**	(2.28, 3.21)**	(1.65, 2.38)**
2005–2006	1.39	2.15	1.39	2.40	1.03	1.61	2.76	1.97
	(1.29, 1.48)**	(1.98, 2.34)**	(1.29, 1.50)**	(2.19, 2.62)**	(0.91, 1.18)	(1.41, 1.85)**	(2.36, 3.24)**	(1.66, 2.34)**
2006–2007	1.36	2.15	1.35	2.37	1.07	1.68	2.76	1.97
	(1.28, 1.45)**	(1.98, 2.33)**	(1.26, 1.45)**	(2.17, 2.58)**	(0.95, 1.21)	(1.48, 1.91)**	(2.38, 3.21)**	(1.68, 2.32)**
2007–2008	1.34	2.14	1.32	2.37	1.07	1.67	2.72	2.00
	(1.26, 1.42)**	(1.98, 2.31)**	(1.24, 1.41)**	(2.18, 2.57)**	(0.96, 1.20)	(1.48, 1.88)**	(2.36, 3.13)**	(1.71, 2.33)**
2008–2009	1.33	2.12	1.32	2.37	1.07	1.63	2.69	1.95
	(1.26, 1.41)**	(1.97, 2.28)**	(1.24, 1.40)**	(2.18, 2.56)**	(0.96, 1.19)	(1.45, 1.83 **	(2.35, 3.08)**	(1.68, 2.26)**
2009–2010	1.32	2.07	1.30	2.31	1.1	1.61	2.71	1.86
	(1.25, 1.40)**	(1.93, 2.22)**	(1.23, 1.38)**	(2.14, 2.49)**	(0.99, 1.21)	(1.44, 1.80 **	(2.38, 3.08 **	(1.61, 2.16)**

Reference group: Non-Aboriginal population. Adjusted for sex (male, female), age group (35–44 years, 45–54 years, 55–64 years, 65–74 years, 75 years and over), socioeconomic status proxy (full subsidy, partial subsidy, no subsidy), area of residence (urban rural, remote)

95% CI = 95% confidence interval; COPD = chronic obstructive pulmonary disease; PR = prevalence ratios

** p<0.001

After adjusting for covariates (Table 3), all annual PRs were significantly higher for all Aboriginal groups compared to the reference group. Compared to the non-Aboriginal group, Registered First Nations were between 2.3 and 2.4 times more likely to have COPD from 2002 to 2010, followed by the Inuit (1.86 to 2.10 times more likely) and the Métis (1.59 to 1.67 times more likely).

**Fig 3:**
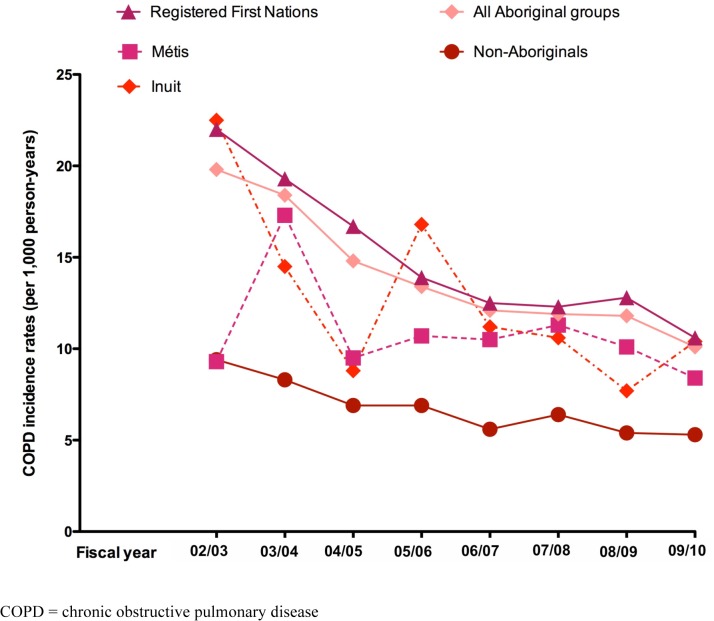
Annual age- and sex-standardized COPD incidence rates in Aboriginal peoples and the non-Aboriginal population in Alberta, Canada from fiscal years 2002 to 2010.

A total of 3,885 new cases of COPD were identified over the 8-year study period. Standardized COPD incidence rates showed annual fluctuations over time, particularly among the Inuit and Métis groups (Fig 3). The standardized COPD incidence density rate for the entire study period in the Aboriginal peoples cohort combined was 11.3 cases per 1,000 person-years (95% CI: 11.2, 11.4/1,000 person-years), which doubled that of the non-Aboriginal group (5.5; 95% CI: 5.4, 5.6/1,000 person-years). Standardized COPD incidence density rates of the three Aboriginal groups were all higher than those of the non-Aboriginal group, with First Nations having the highest COPD incidence density rates (12.3; 95% CI: 12.1, 12.4/1,000 person-years) followed by the Inuit (10.1; 95% CI: 9.7, 10.5/1,000 person-years) and the Métis (8.6; 95% CI: 8.3, 8.8/1,000 person-years).

For every fiscal year, Aboriginal peoples, as a whole group, had significantly higher unadjusted IRR of COPD than the non-Aboriginal group (Table 4). When Aboriginal subgroups were compared to the non-Aboriginal group, we found that the unadjusted COPD IRRs were significantly higher for the Inuit and First Nations only. After adjusting for age, sex, SES and area of residence, all Aboriginal groups had significantly higher COPD IRRs compared to the non-Aboriginal group for every study year.

**Table 4:** Adjusted and unadjusted incidence rate ratios (IRR) of COPD among Aboriginal groups in Alberta (Canada) from fiscal years 2002 to 2010.

Fiscal year	All aboriginal groups		First Nations		Métis		Inuit	
	COPD IRR (95% CI)		COPD IRR (95% CI)		COPD IRR (95% CI)		COPD IRR (95% CI)	
	Unadjusted	Adjusted	Unadjusted	Adjusted	Unadjusted	Adjusted	Unadjusted	Adjusted
2002–2003	1.35	2.11	1.39	2.39	0.82	1.24	2.84	2.22
	(1.13, 1.61)**	(1.73, 2.57)**	(1.16, 1.68)**	(1.94, 2.95)**	(0.57, 1.18)	(0.86, 1.80)	(1.93, 4.18)**	(1.49, 3.30)**
2003–2004	1.30	2.06	1.34	2.27	0.84	1.35	2.57	2.09
	(1.09, 1.55)**	(1.68, 2.52)**	(1.11, 1.62)**	(1.83, 2.82)**	(0.58, 1.21)	(0.93, 1.96)	(1.70, 3.89)**	(1.37, 3.19)**
2004–2005	1.42	2.14	1.50	2.50	1.00	1.44	1.87	1.42
	(1.18, 1.71)**	(1.74, 2.63)**	(1.23, 1.82)**	(2.00, 3.12)**	(0.70, 1.43)	(1.00, 2.08)**	(1.12, 3.12)**	(0.85, 2.39)
2005–2006	1.29	1.87	1.23	2.03	1.10	1.40	3.09	2.04
	(1.07, 1.54)**	(1.53, 2.29)**	(1.01, 1.50)**	(1.62, 2.53)**	(0.79, 1.53)	(1.00, 1.96)	(2.08, 4.58)**	(1.37, 3.06)**
2006–2007	1.41	2.15	1.34	2.36	1.41	1.85	2.67	1.77
	(1.16, 1.71)**	(1.73, 2.68)**	(1.09, 1.65)**	(1.86, 2.99)**	(1.02, 1.95)**	(1.32, 2.58)**	(1.68, 4.2)**	(1.11, 2.84)**
2007–2008	1.36	2.06	1.36	2.43	1.10	1.36	2.47	1.72
	(1.14, 1.62)**	(1.69, 2.05)**	(1.12, 1.64)**	(1.96, 3.01)**	(0.79, 1.53)	(0.97, 1.90)	(1.59, 3.83)**	(1.10, 2.68)**
2008–2009	1.51	2.22	1.56	2.66	1.14	1.43	2.30	1.60
	(1.26, 1.81)**	(1.82, 2.71)**	(1.29, 1.88)**	(2.15, 3.30)**	(0.82, 1.59)	(1.01, 2.00)**	(1.44, 3.69) **	(0.99, 2.58)
2009–2010	1.45	2.01	1.41	2.25	1.36	1.53	2.70	1.76
	(1.21, 1.74)**	(1.64, 2.45)**	(1.17, 1.71)**	(1.81, 2.80)**	(1.00, 1.86)	(1.12, 2.11)**	(1.74, 4.20)**	(1.12, 2.11)**
Incidence density rate 2002–2010	1.48	2.11	1.52	2.37	1.08	1.49	2.54	1.92
	(1.39, 1.58)**	(1.97, 2.27)**	(1.42, 1.63)**	(2.19, 2.56)**	(0.96, 1.22)	(1.32, 1.69)**	(2.18, 2.96)**	(1.64, 2.25)**

Reference group: Non-Aboriginal population. Adjusted for sex (male, female), age group (35–44 years, 45–54 years, 55–64 years, 65–74 years, 75 years and over), socioeconomic status proxy (full subsidy, partial subsidy, none), area of residence (urban rural, remote)

95% CI = 95% confidence interval; COPD = chronic obstructive pulmonary disease; IRR = incidence rate ratios

** p<0.05

The pattern of differences relative to the non-Aboriginal groups on the annual COPD incidence rates over time was not equal for all three Aboriginal groups. Registered First Nations had between 2 to 2.66 times more incident cases of COPD per 1,000 person-years than the non-Aboriginal group, and all annual COPD incidence rates were significantly different over the study period. The Inuit had between 1.42 to 2.22 times more incident cases of COPD per 1,000 person-years than the non-Aboriginal group; however, differences between groups were not significant for some years (2004 to 2005 and 2008 to 2009). The Métis had between 1.2 to 1.85 times more incident COPD cases per 1,000 person-years than the non-Aboriginal group; however, differences were not significant for the first two years of the study and for the year 2007.

**Fig 4:**
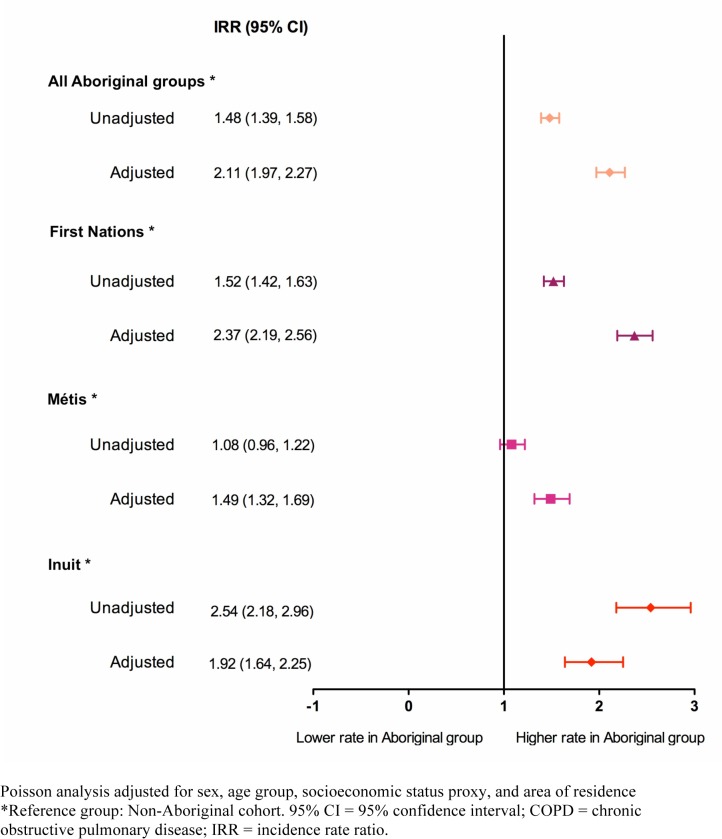
Unadjusted and adjusted COPD incidence rate ratios for Aboriginal groups for the entire study period 2002 to 2010.

After the IRR of COPD was adjusted for important sociodemographic factors (Fig 4), we found that all Aboriginal peoples had a significantly higher number of new COPD cases than the non-Aboriginal group (IRR 2.1; 95% CI: 1.97, 2.27). Compared to non-Aboriginals, the number of COPD incident cases was higher among First Nations (IRR 2.37; 95% CI: 2.19, 2.56) followed by the Inuit (IRR 1.92; 95% CI: 1.64, 2.25) and the Métis (IRR 1.49; 95% CI: 1.32, 1.69).

## Discussion

This is the first study to provide a comprehensive longitudinal assessment of the epidemiology of COPD in the three Aboriginal groups of Canada compared to a non-Aboriginal reference group. Using a validated algorithm for case identification and adjusting for important sociodemographic factors, we found that the three Aboriginal groups had a higher prevalence and incidence of COPD than the non-Aboriginal population. There were differences in the burden of COPD across the three Aboriginal groups, with Registered First Nations and Inuit having the highest COPD rates followed by the Métis. These results are broadly in line with other unpublished longitudinal Canadian studies reporting a higher prevalence and incidence of COPD in specific Aboriginal groups (i.e., Métis) [6–8].

The increased epidemiology of COPD among Aboriginal peoples in our study is likely explained through multiple mechanisms. First, smoking is the most important etiologic factor in the development of COPD [20,21]. The administrative data used in this study, while robust, did not contain smoking history or pack years. Smoking rates in Canada’s Aboriginal populations are, on average, twice as high as those of non-Aboriginal Canadians (~21%), with higher rates for the Inuit (~49%), followed by First Nations (~40%) and Métis (~37%) compared to non-Aboriginals [22]. Therefore, it is reasonable to expect that epidemiological indicators of COPD would be higher in Aboriginal peoples compared to the non-Aboriginal population.

Similarly, exposure to environmental contaminants derived from biomass fuel burned for cooking, and living in poorly ventilated areas can also increase the risk of COPD among certain Aboriginal groups who follow a traditional lifestyle or live on reserves [23]. Interactions between smoking, housing conditions and crowding, material and social deprivation [24], remote location of residence [25], low education [26], poor nutrition, and prenatal and childhood exposures to cigarette smoking are likely to be distal and intermediate mechanisms for the higher epidemiological indicators of COPD burden among Aboriginal peoples in our study.

This study has several limitations. The process of diagnosing COPD is complex and imperfect. While the diagnosis of COPD was not clinically confirmed, the algorithm for case identification has been shown to be valid and have high accuracy [15]. To conduct a similar sized clinical study would require an enormous funding commitment and many years of research compared to efficiency of using administrative databases. Superficial clinical details in administrative databases precluded the acquisition of information on key clinical and sociodemographic confounding variables (e.g., smoking status, smoking history, body mass index, dietary intake, exercise, etc.) to adjust the baseline risk for COPD in multivariate analyses.

An important strength of this study was the comprehensive methods to identify individuals in the Aboriginal cohorts. This is one of the first studies of its kind in Canada that identified Registered First Nations, Métis and Inuit within an Aboriginal cohort, thus reducing the impact of misclassification bias related to the definition of Aboriginal status. Other studies assessing the health status of Aboriginal peoples in Canada [27–30] have not included Inuit and Métis populations in their analyses. Limitations encountered in similar studies, however, persisted into a lesser degree as approximately one-third of Aboriginal peoples in the province may have been undercounted. For example, an individual classified in the non-Aboriginal group may have been in fact, a non-Registered First Nations person or a Métis without citizenship registration (approximately 70% of the Métis population in the province). We acknowledge the designation of Aboriginal groups is imperfect, and these numbers likely under-estimate those Albertans who refer to themselves as Aboriginal.

Finally, the cohort design with linkage of a variety of provincial administrative health databases involved a large number of people with wide coverage and continuity of data over a relatively long follow-up period. It is within reason to expect that our results can be generalized to Aboriginal peoples in Alberta and allow inferences that can be applied to Aboriginal populations in other Canadian provinces.

Future epidemiological research should help to improve our understanding of how Aboriginal status intersects with other social determinants of health to create inequitable conditions associated with a higher risk of COPD. Distinctions in the origin, form and impact of these determinants and how they affect distinctly Aboriginal peoples groups will have powerful implications for health services policy and planning. The incorporation of an equity lens within the existing respiratory health research agenda is an important contribution to the epidemiological study of respiratory diseases, and a great opportunity to start addressing health inequalities pertaining to respiratory health status that affect Aboriginal peoples and other vulnerable groups in our society.

## Conclusions

This study is one of the first to evaluate the epidemiology of COPD among Aboriginal peoples of Canada over an extended period of time and using methods that allowed the identification of Registered First Nations peoples, Métis and Inuit within the Aboriginal cohort. The study demonstrated the existence of a large gap in the prevalence and incidence of COPD affecting Aboriginal populations compared to their non-Aboriginal counterparts in an industrialized country like Canada. Results of this research are important for the planning of respiratory health services delivered to Aboriginal peoples in industrialized countries and to motivate further evaluations of the determinants and pathways of COPD-related inequalities.
